# Effects of fermented *Arctium lappa L. root* by *Lactobacillus* casei on hyperlipidemic mice

**DOI:** 10.3389/fphar.2024.1447077

**Published:** 2024-10-28

**Authors:** MingJu Chen, Yuxiao Wu, Hongxuan Yang, Tianfeng Liu, Tongkun Han, Wangqiang Dai, Junyue Cen, Fan Ouyang, Jingjing Chen, Jianxin Liu, Lin Zhou, Xuguang Hu

**Affiliations:** ^1^ School of Traditional Chinese Medicine, Guangdong Pharmaceutical University, Guangzhou, Guangdong, China; ^2^ Guangdong Provincial Key Laboratory of Advanced Drug Delivery, Guangdong Provincial Engineering Center of Topical Precise Drug Delivery System, School of Life Sciences and Biopharmaceutics, Guangdong Pharmaceutical University, Guangzhou, Guangdong, China; ^3^ Shenzhen Bao’an District Songgang People’s Hospital, Shenzhen, Guangdong, China; ^4^ School of Pharmaceutical Sciences, China-Pakistan International Science and Technology Innovation Cooperation Base for Ethnic Medicine Development in Hunan Province, Hunan University of Medicine, Huaihua, Hunan, China

**Keywords:** Arctium lappa *L. root*, *lactobacillus*, fermented, hyperlipidemia, lipid level, gut microbiota

## Abstract

**Introduction:**

This study aimed to establish a fermentation system based on *Lactobacillus casei* (LC) and *Arctium lappa L. root* (AR) to investigate its effects. The objectives included comparing metabolite profiles pre- and post-fermentation using untargeted metabolomics and evaluating the impact of LC-AR in high-fat diet-induced hyperlipidemic mice.

**Methods:**

Untargeted metabolomics was used to analyze differences in metabolites before and after fermentation. *In vitro* antioxidant activity, liver injury, lipid levels, pro-inflammatory cytokine levels, and cholesterol-related mRNA expression were assessed. 16S rRNA sequencing was conducted to evaluate changes in gut microbiota composition.

**Results:**

LC-AR exhibited stronger antioxidant activity and higher metabolite levels than AR. It also improved liver injury as well as better regulation of lipid levels, pro-inflammatory cytokine levels, and cholesterol-related mRNA. 16S rRNA analysis revealed that LC-AR decreased the Firmicutes/Bacteroidetes ratio, which correlated negatively with triglycerides, total cholesterol, and low-density lipoprotein cholesterol levels.

**Discussion:**

These findings suggest that LC-AR may serve as a promising functional food and drug raw material for improving hyperlipidemia, particularly through its beneficial effects on gut microbiota and lipid regulation.

## 1 Introduction

Hyperlipidemia is a metabolic illness characterized by excessive lipid levels and is a key risk factor for stroke and cardiovascular diseases (Crea, 2023; Alloubani et al., 2021; Stewart et al., 2020) Multiple clinical studies have proved that statins and fibrates exhibit good performance in reducing blood lipids and even the incidence of stroke and cardiovascular diseases. However, their side-effects, such as muscle soreness, liver damage, and renal toxicity, make them unsuitable for long-term use (Alloubani et al., 2021; Wang et al., 2021). Therefore, it is necessary to find a mild alternative therapy with low side effects.

*Arctium lappa L.* (AL) is a herb of the genus Arctium in the Asteraceae family, which is a special plant of medicinal edible homology and an important member of a variety of traditional Chinese medicine formula (Nam et al., 2021; Song et al., 2018). AL exhibits anti-inflammatory (Zhang H. L. et al., 2020), anti-viral (Dias et al., 2017), and anti-constipation (Takeda et al., 1992) properties. Each part of AL has different pharmacological effects. Particullarly, the root of AL, *A. lappa Lactobacillus root* (AR), is rich in polysaccharides (Gao et al., 2023), polyphenols (Moro et al., 2020), flavonoids, and other bioactive components, and has underlying research significance. Past studies have shown that the ethanolic extract of AR can ameliorate obesity and liver steatosis in rats (Ma et al., 2022). In addition, the distilled water extract of AR can regulate lipid profile levels and ameliorate atherosclerotic symptoms in high fat diet-induced quail atherosclerosis (Wang et al., 2016). These results indicate that AR performs well in ameliorating lipid profile levels. However, no studies have yet revealed the effects of AR on improving hyperlipidemia and its specific mechanisms.

*Lactobacillus*, as a probiotic, is present in essential organs, such as the respiratory and gastrointestinal tracts of humans and other mammals, playing a role in regulating microbial community homeostasis (Un-Nisa et al., 2022). It plays an important role in the microbiota of mammals and even ecosystems. It's well known, improving the dysbiosis of gut microbiota can serve as a crucial mechanism for preventing and treating hyperlipidemia caused by lipid metabolism disorders (Liu et al., 2023). Furthermore, *Lactobacillus* has been proven to ameliorate symptoms such as elevated cholesterol and hyperlipidemia in mice (Liu et al., 2020; Chai et al., 2022). Thus, there is a good prospect to expand the application of *lactobacillus*.

Fermentation is one of the methods used in processing traditional Chinese medicine, which has the characteristics of reducing toxicity and enhancing curative effects. In contrast to traditional fermentation technology, modern fermentation technology is mostly probiotic fermentation with Chinese herbs, offering advantages such as well-defined fermentation substrate, controllable processes, and optimization possibilities (Yang et al., 2023). Fermentation technology has great potential in the field of biomedicine, especially in the development of traditional Chinese medicine and modern pharmaceuticals (Diez-Ozaeta and Astiazaran, 2022). China’s traditional fermented medicine, Massa Medicata Fermentata, has a long history and is widely used in clinical practice as a digestive agent, with the functions of strengthening the spleen and gut and promoting digestion (Fu et al., 2020).

Of particular interest is the potential of traditional Chinese herbal fermentation and its apparent efficacy in improving hyperlipidemia. For instance, another traditional fermented Chinese medicine, Monascus purpureus Went, has been clinically proven to significantly reduce LDL-C, total cholesterol, and triglyceride levels, and was well tolerated in patients with hyperlipidemia (Lin et al., 2005). Huang et al. (2019) found that the theabrownin in fermented Pu-erh tea alleviates hypercholesterolemia by modulating the gut microbiota, suggesting that the reduction of gut bile-salt hydrolase (BSH) microbes and/or decreased FXR-FGF15 signaling could be potential therapeutic mechanisms for treating hypercholesterolemia and hyperlipidemia. Meanwhile, Zheng Y. et al. (2024) demonstrated that compared to Bergamot, only fermented Bergamot (Laoxianghuang) could decrease the content of isobutyric acid and isovaleric acid, which are detrimental to gut health, making it more advantageous in improving the gut microbiota of patients with hyperlipidemia. In our recent research, the fermentation of *Lactobacillus acidophilus* and dandelion was found to improve symptoms in mice with hyperuricemia (Ma et al., 2023). Based on this, traditional Chinese fermented medicines seem to be an effective potential supplementary treatment for hyperlipidemia.

Overall, the present study harnesses the advantages of traditional Chinese medicinal herbs, probiotics, and fermentation systems using AR as a fermentation substrate, with *Lactobacillus* as the added strain for liquid fermentation. From various perspectives, including lipid levels, inflammatory factor levels, and gut microbiota composition, we explore the impact and mechanisms of *Lactobacillus* fermentation of AR on hyperlipidemic mice. Investigate its potential in improving hyperlipidemia, providing a theoretical foundation for the expansion of related fields.

## 2 Methods and materials

### 2.1 Materials

*Lactobacillus acidophilus GDMCC1.412* (LA), *Lactobacillus casei GDMCC1.159* (LC), and *Lactobacillus plantarum GDMCC1.191* (LP) were obtained from Guangdong Microbial Center (Guangzhou, China). *Lactobacillus rhamnosus ATCC7469* (LR) and De Man, Rogosa, and Sharpe (MRS) medium were purchased from Huankai Biotechnology Co., Ltd. (Zhaoqing, China). AR was purchased from Guangdong Pharmaceutical University, Zhenpin Yaozhuang. Cholesterol esterase (CEase), sodium taurocholate, p-NPB, Folin-Ciocalteu reagent, 2, 2′-azinobis (3-ethylbenzothiazoline-6-sulfonic acid) (ABTS), 1,1-diphenyl-2-picrylhydrazyl (DPPH), simvastatin, and sodium carboxymethyl cellulose were purchased from Macklin Technology Co., Ltd. (Shanghai, China). Pancreatic lipase (PL) was purchased from Meilun Biotechnology Co., Ltd. (Dalian, China), and the gallic acid standard was purchased from Yuanye Biotechnology Co., Ltd. (Shanghai, China).

2-Chloro-L-phenylalanine was purchased from Shanghai Aladdin Biochemical Technology Co.,Ltd. (Shanghai, China). Total cholesterol (TC), triglyceride (TG), high-density lipoprotein cholesterol (HDL-C), Low-density lipoprotein cholesterol (LDL-C), aspartate transaminase (AST), and alanine amioTransferase (ALT) kits were purchased from Nanjing Jiancheng Reagent Co., Ltd. (Nanjing, China). Tumor necrosis factor receptor-α (TNF-α) and interleukin-6 (IL-6) Elisa kits were purchased from Jiangsu Enzymatic Immunization Co., Ltd. (Nanjing, China).

### 2.2 Preparation of fermented AR

AR was crushed and sifted through a 50-mesh sieve, and then mixed with the MRS medium at a liquid-to-material ratio of 1:10 (v/v). The resulting mixture (AR-MRS) was sterilized at 121°C for 20 min and subsequently cooled to ambient temperature. LA, LC, LP, and LR were grown in MRS broth at 37°C for 48 h, and the above passage procedure was repeated once. Four types of activated *Lactobacillus* (LA, LC, LP, and LR) were used as the fermentation seed liquids. *Lactobacillus* was inoculated into the AR-MRS solution at a seeding volume of 5% (v/v) and fermented at 37°C for 96 h. The resulting fermentation liquid was centrifuged at 8,000 rpm for 10 min, and the supernatant was collected as the final product (LA-AR, LC-AR, LP-AR, and LR-AR) (Ma et al., 2023).

### 2.3 Determination of *in vitro* hypolipidemic activity

#### 2.3.1 Determination of cholesterol esterase activity

For determining the activity of cholesterol esterase (CEase), Zhang X. et al. (2020) method was used with minor modification. CEase solution configuration: An appropriate amount of CEase was dissolved in sodium phosphate buffer with a pH of 7.0 to obtain a CEase solution with a final concentration of 1.05 U/mL.

p-NPB solution configuration: An appropriate amount of sodium taurine cholate was dissolved in sodium phosphate buffer with a pH of 7.0 to obtain a sodium taurine cholate solution with a final concentration of 5.16 mM. An appropriate amount of p-NPB was dissolved in 5.16 mM sodium taurine cholate solution to obtain a p-NPB solution with a final concentration of 1 mM.

Sample measurement: 50 μL CEase solution (1.05 U/mL), 50 μL phosphate buffer, and 50 μL sample solution with different concentrations (5, 10, 15, 20, 25, and 30 mg/mL) were mixed evenly and added to a 96-well plate and incubated at 37°C in a thermostatic incubator for 10 min. Then, 50 μL of p-NPB solution was added to the mixture, mixed evenly, and incubated for 5 min, and the absorbance value of the above mixture was measured at 405 nm. The final result was expressed as IC50. The inhibition rate of CEase activity was calculated as follows:

$$\text{Inhibition rate }\left(\%\right)=\left[1‐\;\left(A_{1}\;‐\;A_{2}\right)/\;\left(A_{3}\;‐A_{4}\right)\right]\times 100\%$$ (1)

A_1_: absorbance value of the sample, A_2_: absorbance value of the enzyme replaced with an equal volume buffer solution, A_3_: absorbance value of the sample replaced with an equal volume buffer solution, A_4_: Absorbance value of the sample and enzyme replaced with an equal volume buffer solution.

#### 2.3.2 Determination of pancreatic lipase activity

For determining the activity of pancreatic lipase (PL), Zhang Q. et al. (2020) method was used with minor modifications. PL solution configuration: An appropriate amount of PL was dissolved in sodium phosphate buffer with a pH of 7.0 to obtain a PL solution with a final concentration of 6 mg/mL.

p-NPB solution configuration: An appropriate amount of p-NPB was dissolved in sodium phosphate buffer with a pH of 7.0 to obtain a p-NPB solution with a final concentration of 1 mM.

Sample measurement: 50 μL PL solution (6 mg/mL), 50 μL phosphate buffer, and 50 μL sample solution with different concentrations (5, 10, 15, 20, 25, and 30 mg/mL) were mixed evenly, added to the 96-well plate, and incubated at 37°C in the thermostatic incubator for 10 min. Then, 50 μL of the p-NPB solution was added to the mixture, mixed evenly, and incubated for 5 min, and the absorbance value of the above mixture was measured at 405 nm. The final result was expressed as IC50. The inhibition rate of PL enzyme activity was calculated as follows:

$$\text{Inhibition rate }\left(\%\right)=\left[1‐\;\left(A_{a}\;‐\;A_{b}\right)/\;\left(A_{c}\;‐A_{d}\right)\right]\times 100\%$$ (2)

A_a_: absorbance value of the sample, A_b_: absorbance value of the enzyme replaced with an equal volume buffer solution, A_c_: absorbance value of the sample replaced with an equal volume buffer solution, A_d_: Absorbance value of the sample and enzyme replaced with an equal volume buffer solution.

### 2.4 Determination of phenolic contents

The phenolic content was determined in accordance with the method described in Lyu et al.’s paper (Lyu et al., 2020) with minor modifications. First, 0.1 mL of the sample solution was mixed with 0.2 mL Folin-Ciocalteu reagent and shaken well. Then, 0.4 mL of 12% Na2CO3 was added to the mixture, to which 4.3 mL of distilled water was added and the volume was adjusted to 5 mL. The mixture was incubated at ambient temperature in the dark for 1 h. The absorbance of the resulting mixture was measured at 765 nm. The total phenolic content was expressed as the Gallic acid equivalent (mg GAE/g).

### 2.5 *In vitro* antioxidant activity

#### 2.5.1 Scavenging capacity of DPPH• free radicals

The scavenging capacity of DPPH• free radicals was determined in accordance with Li Z. et al. (2018) method with simple modifications. A 2 mL DPPH solution (0.1 mg/mL) was mixed with 1 mL of the sample and incubated in the dark for 30 min. The absorption value of the mixture was measured at 517 nm. Equation 3 was used to calculate the DPPH• free radicals’ scavenging capacity for each sample:

$$\text{DPPH}•\text{ scavenging capacity }\left(\%\right)=\left[\left(A_{0}\;‐\;A_{S}\right)/\;A_{0}\right]\times 100\%$$ (3)

A_0_: absorbance value of equal volume anhydrous ethanol instead of the sample, A_S_: absorption value of the sample mixed with DPPH solution.

#### 2.5.2 Scavenging capacity of ABTS + free radicals

The ABTS + free radicals’ scavenging capacity was determined according to Li Z. et al. (2018) method with simple modifications. A 2 mL ABTS solution was mixed with 1 mL of the sample and incubated in the dark for 6 min. The absorption value of the mixture was measured at 734 nm. Equation 4 was used to calculate the ABTS + free radicals’ scavenging capacity for each sample:

$$\text{ABTS}+\text{scavenging capacity }\left(\%\right)=\left[\left(A_{0}\;‐\;A_{Z}\right)/A_{0}\right]\times 100\%$$ (4)

A_0_: absorbance value of equal volume distilled water instead of the sample, A_Z_: absorption value of the sample mixed with ABTS solution.

### 2.6 Untargeted metabolomics analysis

Metabolite Sample Preparation: transfer an appropriate amount of fermentation broth into a centrifuge tube, concentrate it to dryness, and then dissolve it with 500 µL of methanol solution. Centrifuge the mixture at 12,000 rpm and 4°C for 10 min. Collect the entire supernatant and dry it. Subsequently, dissolve the sample in 150 µL of 2-Chloro-L-phenylalanine solution (prepared in 80% methanol-water). Filter the solution through a 0.22 µm microporous membrane to obtain the sample for analysis (Dunn et al., 2011).

An untargeted metabolomics analysis was conducted using the Thermo Vanquish ultra-high-performance liquid chromatography–tandem mass spectrometry (UPLC-MS/MS) (Thermo Fisher Scientific, United States).

Thermo Vanquish ultra-high-performance liquid chromatography system (Thermo Fisher Scientific, United States). The chromatographic column employed was ACQUITY UPLC^®^ HSST3 (2.1 mm × 150 mm, 1.8 μm) from Waters (Milford, MA, United States).

In the positive ion mode, the mobile phase comprised 0.1% formic acid in acetonitrile (C) and 0.1% formic acid in water (D). The gradient elution program was as follows: 0–1 min, 2% C; 1–9 min, 2%–50% C; 9–12 min, 50%–98% C; 12–13.5 min, 98% C; 13.5–14 min, 98%–2% C; and 14–20 min, 2% C. In the negative ion mode, the mobile phase included acetonitrile (A) and 5 mM ammonium formate in water (B). The gradient elution program was as follows: 0–1 min, 2% A; 1–9 min, 2%–50% A; 9–12 min, 50%–98% A; 12–13.5 min, 98% A; 13.5–14 min, 98%–2% A; and 14–17 min, 2% A. The column temperature was set at 40°C, and the flow rate and injection volume were 0.25 mL/min and 2 μL, respectively (Zelena et al., 2009).

The Q Exactive mass spectrometer (Thermo Fisher Scientific, United States) was used for MS data acquisition. The sheath gas flow rate and auxiliary gas flow rate were set at 30 and 10 arb, respectively. The capillary temperature was maintained at 325°C, with positive and negative ion spray voltages set at 3.50 and −2.50 kV, respectively.

The R package XCMS was used for peak detection, filtering, and alignment, resulting in a comprehensive table of substance quantification. Substance identification was conducted using public databases, such as HMDB (http://www.hmdb.ca) (Wishart, 2020), MassBank (http://www.massbank.jp/) (Horai et al., 2010), LipidMaps (http://www.lipidmaps.org) (Sud et al., 2007), mzCloud (https://www.mzcloud.org) (Abdelrazig et al., 2020), and KEGG (WIXON and KELL, 2000). Ropls software was utilized for all multivariate data analyses, and models were established based on principal component analysis (PCA) and orthogonal partial least squares-discriminant analysis (OPLS-DA).

### 2.7 Animal experiment and design

Fifty-six C57BL/6 male mice, aged 6 weeks, were acquired from Guangdong Zhi-yuan Experimental Animal Co., Ltd., bearing certificate number No.44826500000863. After a week of adaptive feeding, the mice were randomly divided into seven groups (n = 8): the control group (CON); the model group (MOD), which was fed a high-fat diet; the positive group (SIM), which was treated with simvastatin at a dose of 5 mg/kg/d; the low-dose group (AL) treated with AR at a dose of 1 g/kg/d; the high-dose group (AH) treated with AR at a dose of 4 g/kg/d; the low-dose group (LL) treated with AR fermented by LC at a dose of 1 g/kg/d; and the high-dose group (LH) treated with AR fermented by LC at a dose of 4 g/kg/d. The remaining groups were given a diet high in fat, containing 40% high fat, 2% cholesterol, and 0.5% sodium cholate, while the control group followed a regular diet. Each mice group received the drug or saline once per day by tube feeding at a dose of 0.1 mL of fluid per 10 g of body weight. Weekly weight records were maintained. After undergoing different treatments for a duration of 8 weeks, blood samples were collected after a 12-h period of fasting, and subsequently, the serum was frozen at −80°C. After collecting the blood samples, the mice were euthanized using cervical dislocation. The small intestine and liver were meticulously taken and weighed. Prior to the analysis, all these samples were stored at −80°C. The experimental protocol was conducted in accordance with the Regulations for the Management of Laboratory Animals (issued by the National Science and Technology Commission of the People’s Republic of China) and received approval from the Animal Ethics Committee of Guangdong Pharmaceutical University (Approval Number gdpulacspf-2017688).

### 2.8 Serum biochemistry analysis

The serum levels of LDL-C, HDL-C, TG, T-CHO, ALT, and AST were measured using commercial kits (Nanjing, China), and those of IL-6 and TNF-α in the blood were evaluated using a commercial enzyme immunoassay kit (ELISA).

### 2.9 Histology analysis

Following a 24 h fixation in 4% paraformaldehyde, thin slices of the liver were obtained and subsequently embedded in paraffin. Hematoxylin and eosin stained sections were then used to observe the histopathological changes under a Nikon Eclipse Ci-L optical microscope.

### 2.10 Quantitative real-time polymerase chain reaction

RNA was extracted from both the entire liver and the small intestine using Trizol lysate. Before conducting further tests, the RNA concentration was assessed using a nucleic acid analyzer, ensuring purity values ranging from 1.8 to 2.0. Next, the RNA was converted into cDNA using a reverse transcription kit. Finally, the cDNA that was transcribed in the opposite direction, along with a dye that emitted light, was used as reference and inserted into a real-time polymerase chain reaction machine for the response. The average was calculated by repeating each sample three times and correcting it with the internal reference GAPDH. The 2^−ΔΔCT^ method was used to calculate the relative expression of the target gene mRNA. Supplementary Table S1 displays the primer sequences.

### 2.11 16S rRNA analysis

DNA was extracted from the cecal contents using a DNA extraction kit, and the purity of the DNA was checked via 1% agarose gel electrophoresis. The target fragment within the bacterial 16S V3 and V4 regions was selected, and the universal bacterial primers 338F 5′-ACT​CCT​ACG​GGA​GGC​AGC​A-3′ and 806R 5′-GGACTACHVGGGTWTCTAAT-3′ were used as amplification primers for PCR. The PCR products were purified, quantified, and homogenized to create a sequencing library. Then, library QC was performed for constructing libraries. Qualified libraries were sequenced on the Illumina NovaSeq 6000 platform. The high-throughput sequencing was carried out by Beijing Biomarker Technologies Co., LTD. (Beijing, China).

Sequencing result processing:

Raw reads filtration: Raw reads were first filtered by Trimmomatic v0.33. Then, the primer sequences were identified and removed using cutadapt 1.9.1, resulting in high-quality reads without primer sequences.

DADA2 de-noise: De-noising was carried out using the DADA2 method in the R library to remove chimeric sequences, producing non-chimeric reads.

Bioinformatic analysis: This included feature identification (OTUs clustering), diversity analysis, differential analysis, and correlation analysis.

### 2.12 Statistical analysis

The data results are expressed as mean ± SD. GraphPad Prism 8.0 was employed for statistical analysis. Significant differences between the groups were determined using a one-way analysis of variance. A *p*-value < 0.05 was considered statistically significant.

## 3 Results

### 3.1 Analysis of *in vitro* hypolipidemic activity

CEase and PL are jointly involved in the metabolism, digestion, and absorption of dietary cholesterol esters, fat-soluble vitamins, TGs, and other substrates (Mudgil et al., 2021). The targeted inhibition of CEase and PL activities can indirectly improve the symptoms of metabolic disorders, such as obesity and hyperlipidemia. This study established five subgroups to investigate the effects of fermentation, as well as different strains, on fermented AR. The subgroups included a control group (AR) and four experimental groups (LC-AR, LA-AR, LR-AR, and LP-AR). Compared to the control group, the CEase activity in all four experimental groups showed a significant reduction (*p* < 0.001) (Table 1). Especially, the LC-AR group exhibited the most pronounced decrease in CEase activity (*p* < 0.001), with a reduction of 56.70% compared to the AR group. This suggests that the inhibitory effect of AR on CEase is significantly enhanced after fermentation with different bacterial strains, with the LC-AR group demonstrating the strongest inhibitory effect. In addition, compared to the control group (AR), the PL activities in the LC-AR, LA-AR, and LR-AR groups all showed a significant reduction *(p* < 0.001). However, no significant difference in PL activity (*p* > 0.05) was observed in the LP-AR group. Among these, the LR-AR group decreased most markedly in PL activity (*p* < 0.001), with a reduction of 26.60% compared to the AR group.

**TABLE 1:** IC50 of CEase and PL activity among different groups.

Group	Cease (mg/mL)	PL (mg/mL)
AR	8.36 ± 0.60	7.22 ± 0.10
LC-AR	3.62 ± 0.04^a^	5.46 ± 0.03^a^
LA-AR	4.40 ± 0.10^a^ ^,^ ^b^	6.76 ± 0.07^a^ ^,^ ^d^
LR-AR	4.73 ± 0.05^a^ ^,^ ^c^	5.30 ± 0.06^a^
LP-AR	5.91 ± 0.12^a^ ^,^ ^d^	7.08 ± 0.05^d^

In comparison to AR.

^a^ Significant at *p* < 0.001; in comparison to LC-AR.

^b^ Significant at *p* < 0.05.

^c^ Significant at *p* < 0.01.

^d^ Significant at *p* < 0.001. AR: *Arctium lappa L. root;* LC-AR: *Lactobacillus casei-Arctium lappa L. root;* LA-AR: *Lactobacillus acidophilus-Arctium lappa L. root;* LR-AR: *Lactobacillus rhamnosus-Arctium lappa L. root;* LP-AR: *Lactobacillus plantarum-Arctium lappa L. root.*

These findings suggests that the fermentation of AR by different types of *lactobacillus* have varying *in vitro* lipid-lowering effects. This is attributable to the distinct metabolic characteristics and production of different metabolites during the metabolic processes of LA, LC, LP, and LR (Fiorino et al., 2023). Overall, the CEase activities in the LC-AR group were significantly lower than those in the other groups, while the PL activities in both the LC-AR and LR-AR groups were significantly lower than in the other groups, with no significant difference between the two. More importantly, research indicates that LC has a notable regulatory effect on lipid metabolism disorders in hyperlipidemia mice, suggesting its promising potential in improving hyperlipidemia (Qian et al., 2019; Yang D. et al., 2019). Based on the CEase and PL activities of each experimental group, and considering overall better *in vitro* hypolipidemic activity, LC was selected as the experimental strain for the subsequent fermentation of AR.

### 3.2 Analysis of phenolic contents and *in vitro* antioxidant activity

There appears to be a certain correlation between the oxidative stress generated by the accumulation of adipose tissue and the development of metabolic complications (Le et al., 2014). Meanwhile, phenolic compounds exhibit a certain level of antioxidant activity both *in vitro* and *in vivo* (Liu et al., 2022).

Based on the variation in phenolic compound content (Table 2), the LC-AR group (13.17 ± 0.10 mg/g) exhibited a significant increase of 1.08 ± 0.03 times compared to the AR group (6.34 ± 0.15 mg/g) (*p* < 0.001). Simultaneously, the *in vitro* antioxidant capacity of the samples was investigated through DPPH• and ABTS + free radical scavenging experiments. Typically, IC50 can be used to assess the *in vitro* antioxidant capacity of samples. A lower IC50 value indicates that the samples can more efficiently eliminate free radicals at the same equivalent. Interestingly, after fermentation, the clearance rates of the DPPH• free radical (3.11 ± 0.12) and ABTS + free radical (0.61 ± 0.02) of the LC-AR group showed a significant reduction in IC50 values compared to the AR group (*p* < 0.001), by 22.10% and 40.20% times, respectively. This suggests that LC fermentation of AR can increase its total phenolic content and enhance its *in vitro* antioxidant activity.

**TABLE 2:** Phenolic contents and IC50 values of antioxidant activities among different groups.

Group	Phenolic content (mg/g)	DPPH (mg/mL)	ABTS (mg/mL)
AR	6.34 ± 0.15	3.99 ± 0.04	1.02 ± 0.02
LC-AR	13.17 ± 0.10^a^	3.11 ± 0.12^a^	0.61 ± 0.02^a^

^a^ Significant at *p* < 0.001. AR, *Arctium lappa L. root;* LC-AR, *Lactobacillus casei-Arctium lappa L. root*.

### 3.3 Analysis of untargeted metabolomics

Based on the UPLC–MS/MS untargeted metabolomic analysis, the differences in the metabolite composition between AR and LC-AR were assessed. Through comparison of database information, including mass-to-charge ratio, retention time, and peak area, a preliminary identification of 276 compounds was achieved (Supplementary Table S2). These involved 25 phenolic compounds, 37 amino acids and their derivatives, and 21 lipid metabolites.

**FIGURE 1:**
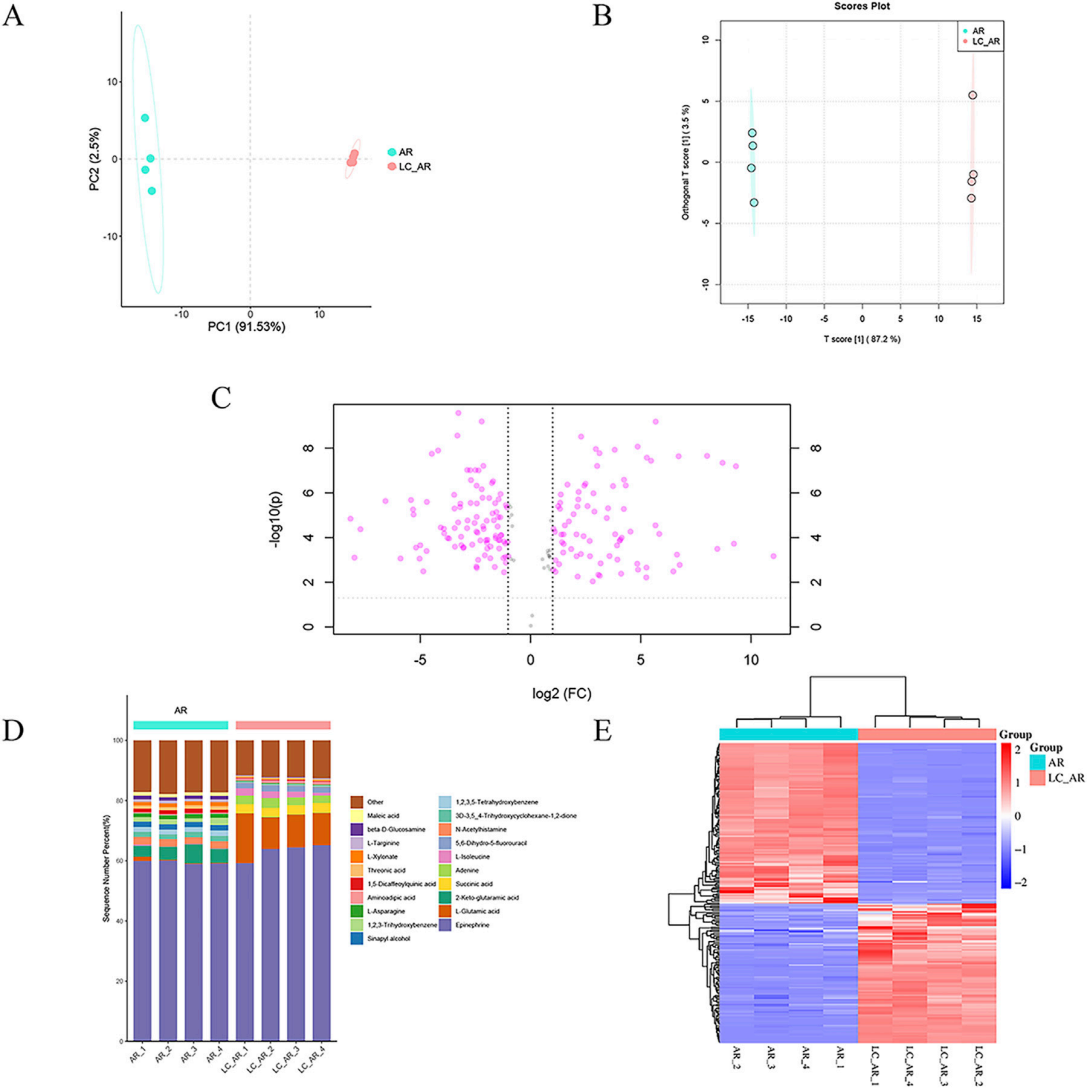
The plot of **(A)** PCA and **(B)** OPLS-DA scores, **(C)** Volcano plot, **(D)** percentage stacked Columns, **(E)** clustered heatmap for AR and LC-AR, n = 4.

Using multivariate statistical techniques, we conducted a macroscopic analysis of the differences in metabolites between AR and LC-AR. Unsupervised PCA was employed for data dimensionality reduction (Figure 1A). The two-dimensional PCA scatter plot formed by the first principal component (PC1) and the second principal component (PC2) revealed a significant separation between the AR and LC-AR groups, indicating substantial differences in the compositions of metabolites between the two groups. Simultaneously, the relatively small separation within each group suggested the stability of the experimental system.

Furthermore, we employed supervised OPLS-DA to enhance the effectiveness of the data analysis. OPLS-DA decomposes the X matrix into two types: information correlated with Y and uncorrelated with Y. By eliminating the uncorrelated data variations, the classification information is concentrated mainly on one principal component, effectively filtering out noise unrelated to the classification information. As shown in Figure 1B, there was a high degree of separation between the AR and LC-AR groups. Simultaneously, in the OPLS-DA model developed in this study, the values of R2X, R2Y, and Q2 were 0.906, 1, and 0.999, respectively, all approaching 1, indicating the model’s effectiveness and rationality. In contrast, the model utilized the value importance in projection (VIP) values and p-values as critical indicators of the extent of metabolite changes. Generally, VIP > 1 and *p* < 0.05 are considered significant differential metabolites, with higher VIP values and lower p-values indicating a greater contribution to the grouping. Based on this, the OPLS-DA model screened 198 significant differential metabolites (Supplementary Table S3).

To visually analyze the differences in metabolite changes between the AR and LC-AR groups, we created three typical metabolomic analysis visualizations—volcano plot, percentage stacked columns, and clustering heatmap (Figures 1C–E)—and compared the data between these two groups. In the volcano plot, the abscissa represents the fold change of metabolites, indicated by log2 (Fold Change, FC), while the ordinate represents the T-test p-value, denoted as -log10 (p). As shown in Figure 1C, each point represents a specific metabolite. Generally, metabolites with -log10 (*p*) > 1.301 and |log2 (FC)| > 1 exhibit significant differences. Compared to the AR group, the LC-AR group showed 79 significantly upregulated metabolites and 97 significantly downregulated metabolites. Percentage stacked columns are formed of the top 20 metabolites based on relative abundance (Figure 1D). Epinephrine is the most abundant metabolite in both the AR and LC-AR groups, constituting the highest percentage content. The second-highest percentage content in the AR and LC-AR groups is found in 2-Keto-glutaramic acid and L-Glutamic acid, respectively. Simultaneously, L-Glutamic acid shows the most significant variation in content among the LC-AR group metabolites. A clustered heatmap (Figure 1E), where red denotes an increase in metabolite content and blue denotes a decrease, also allows us to see the variations in metabolites between the two groups. The heatmap shows a clear separation between the AR and LC-AR groups, which suggests that the two groups’ metabolite contents differ significantly.

### 3.4 Effects on body weight and liver weight in mice with hyperlipidemia

**FIGURE 2:**
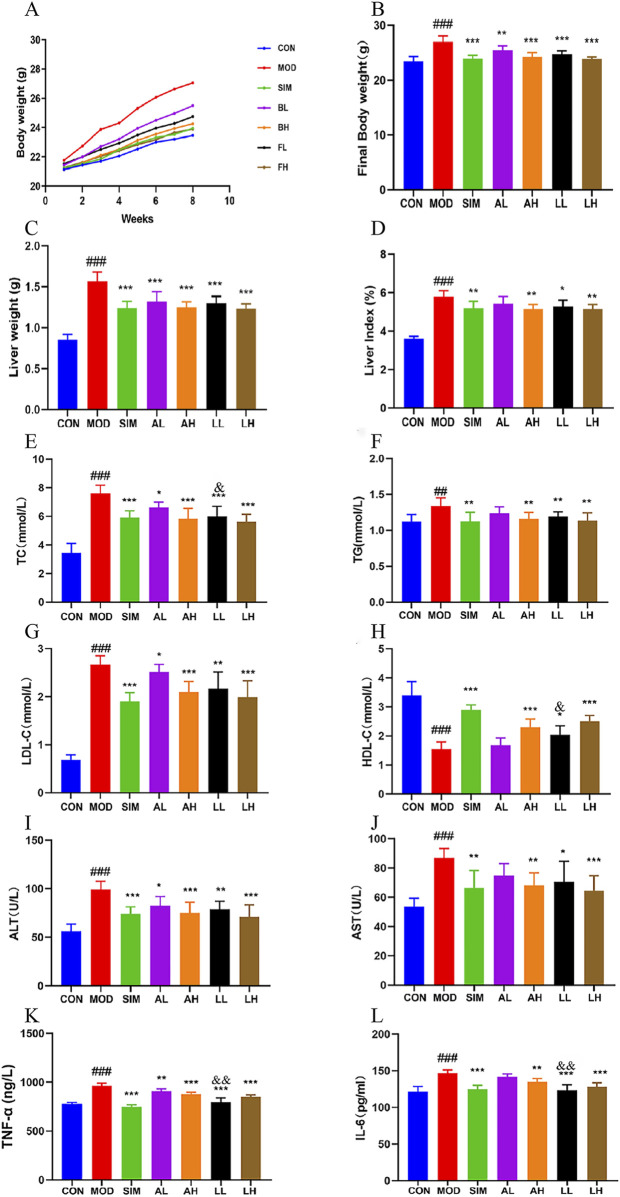
Effects of AR and LC-AR in mice with hyperlipidemia. **(A)** The body weight curve; **(B)** the final weight; **(C)** the weight of the liver; **(D)** The liver index; **(E)** TC level; **(F)** TG level; **(G)** LDL-C level; **(H)** HDL-C level; **(I)** ALT level; **(J)** AST level; **(K)** TNF-α; **(L)** IL-6 level. Data were expressed as mean ± SD, n = 8. Compared with the CON group, ^#^ significant at *p* < 0.05; ^##^ significant at *p* < 0.01; and ^###^ significant at *p* < 0.001; compared with MOD group, ^*^ significant at *p* < 0.05; ^**^ significant at *p* < 0.01; and ^***^ significant at *p* < 0.001; compared with AL group, ^&^ significant at *p* < 0.05.

The hyperlipidemia caused by a long-term high-fat diet is characterized by symptoms such as weight gain and abnormal liver status (Li et al., 2020). The weekly weight fluctuations and final weights of each group of animals are documented in Figures 2A, B. Following 8 weeks of feeding, the body weight of the mice in the high-fat model group significantly rose compared to the control group (*p* < 0.01). The body weight of mice in each treatment group showed a significant decrease compared to the model group (*p* < 0.01).

Compared to the control group, the model group of rats showed a significant increase in liver weight and liver index (*p* < 0.01) (Figures 1C, D). Compared to the model group, the liver weights of the mice in each treatment group showed a significant decrease (*p* < 0.05, *p* < 0.01). The SIM, AH, LL, and LH groups exhibited a highly significant reduction in liver index (*p* < 0.01). However, the AL group showed no significant difference in liver index compared to the model group (*p* > 0.05).

These findings suggest that both AR and LC-AR can effectively alleviate symptoms such as weight gain and liver weight gain induced by hyperlipidemia to a certain extent.

### 3.5 Effect on lipid levels in mice with hyperlipidemia

Dysregulation of lipid levels is a prominent feature of hyperlipidemia (Rauf et al., 2022), and Ha et al. (2018) confirmed that consuming AL can improve serum lipid levels and vascular elasticity in the human body, playing a secondary role in promoting vascular health. The levels of TC, TG, and LDL-C in the model group were significantly elevated compared to the normal group (Figures 2E–H), while HDL-C levels were significantly reduced (*p* < 0.01), indicating the successful establishment of the hyperlipidemic model. Following 8 weeks of treatment, the levels of TC, TG, and LDL-C experienced a significant reduction in the SIM, AH, LL, and LH groups, while the HDL-C level showed a significant increase (*p* < 0.01, *p* < 0.05). However, there were no significant differences in TG and HDL-C between the AL and model groups (*p* > 0.05). In addition, the LL group showed a significant decrease in TC levels and a significant increase in HDL-C levels compared to the AL group (*p* < 0.05). Briefly, both AR and LC-AR can improve lipid-level disorders, with LC-AR being slightly more effective than AR.

### 3.6 Effects on the serum levels AST and ALT levels in hyperlipidemia mice

Lipid-level disorders can further lead to hepatocellular steatosis, ultimately evolving into fatty liver and other complications. Meanwhile, when liver cells are damaged, AST and ALT in the cytoplasm are released into the bloodstream (Chen et al., 2020). According to Figures 2I, J, the levels of AST and ALT in the serum of the model group were considerably elevated compared to the control group (*p* < 0.05). In contrast to the model group, the levels of AST and ALT in the serum of the mice in the SIM, AH, LL, and LH groups showed a significant decrease (*p* < 0.05, *p* < 0.01). Simultaneously, the ALT levels in the AL group showed a significant decrease compared to the model group (*p* < 0.05), while the AST levels did not exhibit a significant difference (*p* > 0.05). Both AR and LC-AR have the potential to improve liver damage caused by hyperlipidemia.

### 3.7 Effects on the levels of TNF-α and IL-6 cytokines levels in hyperlipidemia mice

The establishment of hyperlipidemia models is accompanied by high expression levels of proinflammatory cytokines, which have become potential risk factors for various complications (Soujanya and Jayadeep, 2022). For these pro-inflammatory substances, the model group exhibited significantly higher levels of TNF-α and IL-6 in their serum compared to the normal group (*p* < 0.01) (Figures 2K, L). Compared to the model group, serum TNF-α and IL-6 levels in the SIM, AH, LL, and LH groups were significantly decreased (*p* < 0.01). Compared to the MOD group, the AL group showed a significant decrease in TNF-α levels (*p* < 0.05), while IL-6 levels did not exhibit a significant difference (*p* > 0.05). Meanwhile, the TNF-α and IL-6 levels in the LL group showed a significant decrease compared to the AL group (*p* < 0.01). These findings indicate that both AR and LC-AR demonstrated a certain degree of anti-inflammatory effects, and at low doses, the efficacy of LC-AR appeared to be superior to that of AR.

### 3.8 Effects on liver tissue pathology in hyperlipidemia mice

**FIGURE 3:**
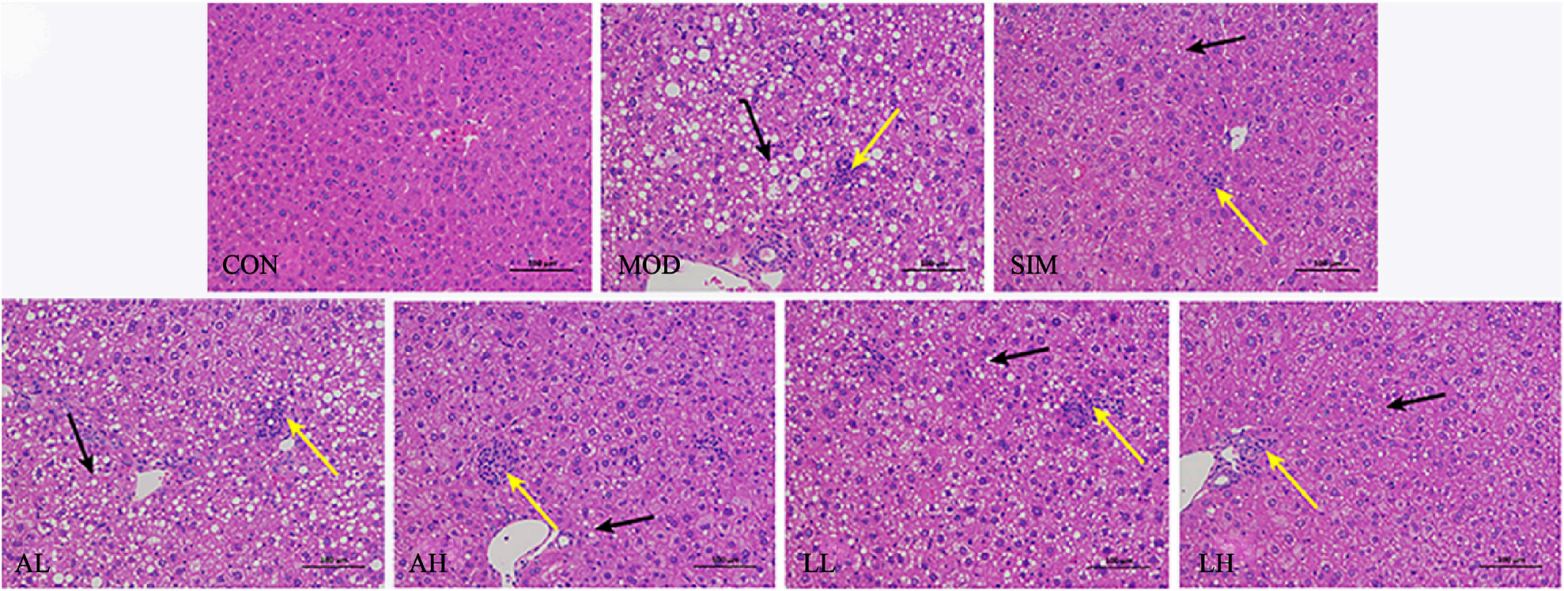
Effect of AR and LC-AR on the pathology of liver tissue. Steatosis is indicated by black arrows, inflammation is indicated by yellow arrows, and the morphology of liver tissue was analyzed using H and E staining at a magnification of 200.

The damage caused by hyperlipidemia includes liver injury and degeneration (Wang et al., 2023). According to Figure 3, the normal group exhibited no signs of fatty degeneration or inflammatory infiltration in liver cells. The liver cells are abundant, arranged orderly, with normal nuclei. However, the mice in the model group exhibited severe swelling of liver cells due to a high-fat diet. Numerous lipid droplets are observed within most liver cells, with some droplets being large and showing evident fatty degeneration and inflammatory infiltration. Furthermore, the mice in the SIM, AH, LL, and LH groups exhibited mild-to-moderate hepatocyte swelling in the liver cells. Simultaneously, there was a reduction in lipid droplet count, and both fatty degeneration and liver cell inflammation infiltration showed significant improvement. In the AL group, there was a certain improvement in inflammatory infiltration of liver cells, but significant lipid droplets were still present, showing no significant deviation from the model group. This suggests that AR and LC-AR can improve liver damage caused by hyperlipidemia.

### 3.9 Effects on mRNA levels related to cholesterol in hyperlipidemia mice

**FIGURE 4:**
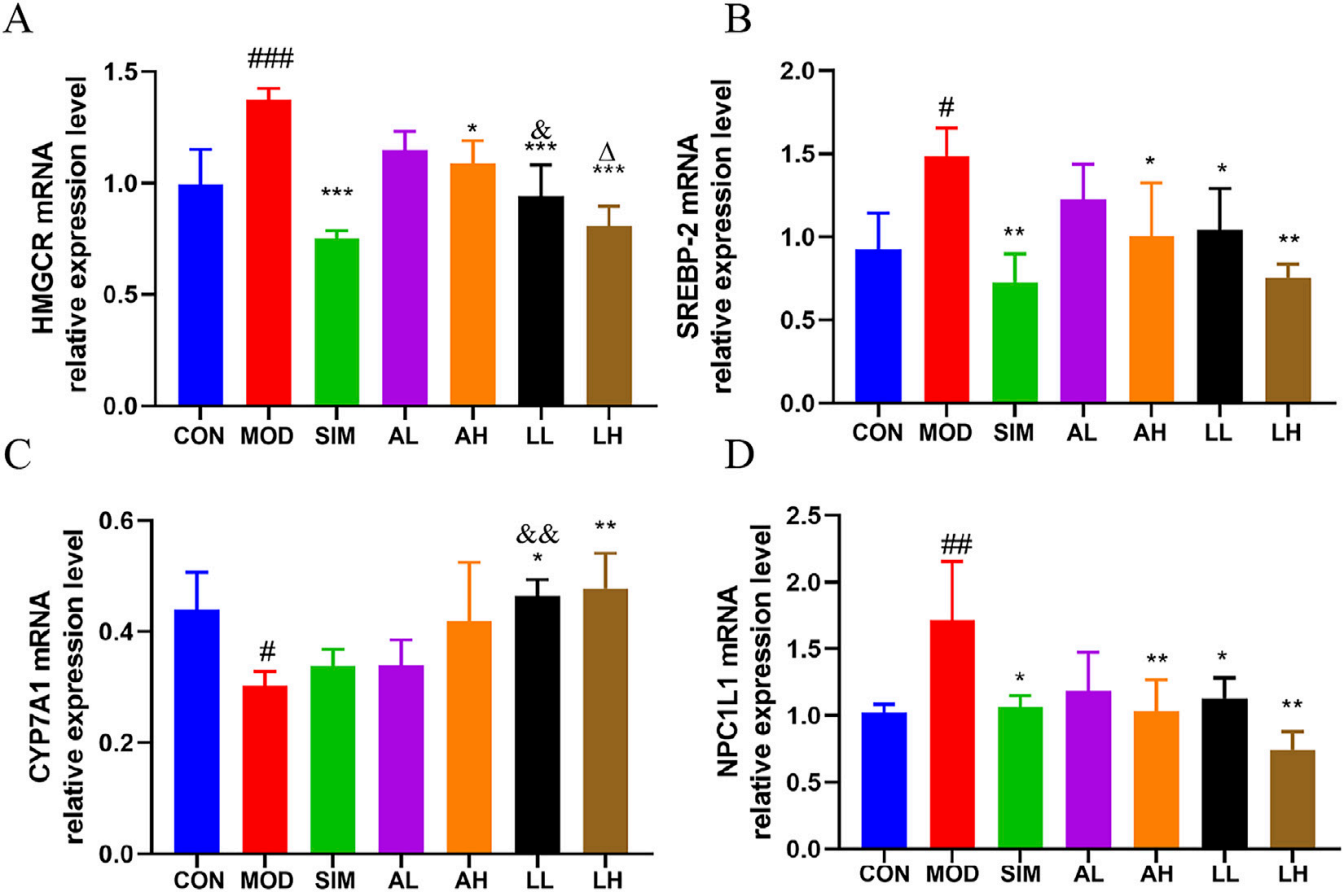
Alterations in gene expression related to cholesterol in the liver and small intestine caused by AR and LC-AR; **(A)** HMGCR in liver; **(B)** SREBP-2 in Liver; **(C)** CYP7A1 in Liver; **(D)** NPC1L1 in small intestine; the results were reported as the mean ± SD, n = 8. Compared the CON group, ^#^ significant at *p* < 0.05, ^##^ significant at *p* < 0.01, and ^###^ significant at *p* < 0.001. In relation to the MOD group, ^*^ significant at *p* < 0.05, ^**^ significant at *p* < 0.01, and ^***^ significant at *p* < 0.001. Compared the AL group, ^&^ significant at *p* < 0.05, and ^&&^ significant at *p* < 0.01. When compared to the AH group, ∆ significant at *p* < 0.05.

We evaluated the expression levels of cholesterol-related mRNA, including HMGCR, SREBP-2, CYP7A1 (liver), and NPC1L1 (small intestine) (Figure 4), to explore the molecular basis of the anti-hyperlipidemic effects of AR and LC-AR. Compared to the control group, the model group showed a significant increase in HMGCR, SREBP-2, and NPC1L1 mRNA levels (*p* < 0.01, *p* < 0.05). When compared to the model group, the AH, LL, and LH groups exhibited a significant decrease in HMGCR, SREBP-2, and NPC1L1 mRNA levels (*p* < 0.01, *p* < 0.05). Meanwhile, the model group had a significant decrease in CYP7A1 mRNA levels (*p* < 0.05), with only the LL and LH groups showing significant increases in CYP7A1 mRNA levels (*p* < 0.01, *p* < 0.05).

Additionally, the HMGCR mRNA levels in the LL group were significantly lower than those in the AL group, while the CYP7A1 mRNA levels were significantly higher than those in the AL group (*p* < 0.05). The results indicate that LC-AR is superior to AR in downregulating hepatic HMGCR and SREBP-2 to inhibit cholesterol synthesis, upregulating hepatic CYP7A1 to promote cholesterol breakdown, and downregulating small intestine NPC1L1 to inhibit cholesterol absorption.

### 3.10 Analysis of gut microbiota in hyperlipidemia mice by alpha diversity

**FIGURE 5:**
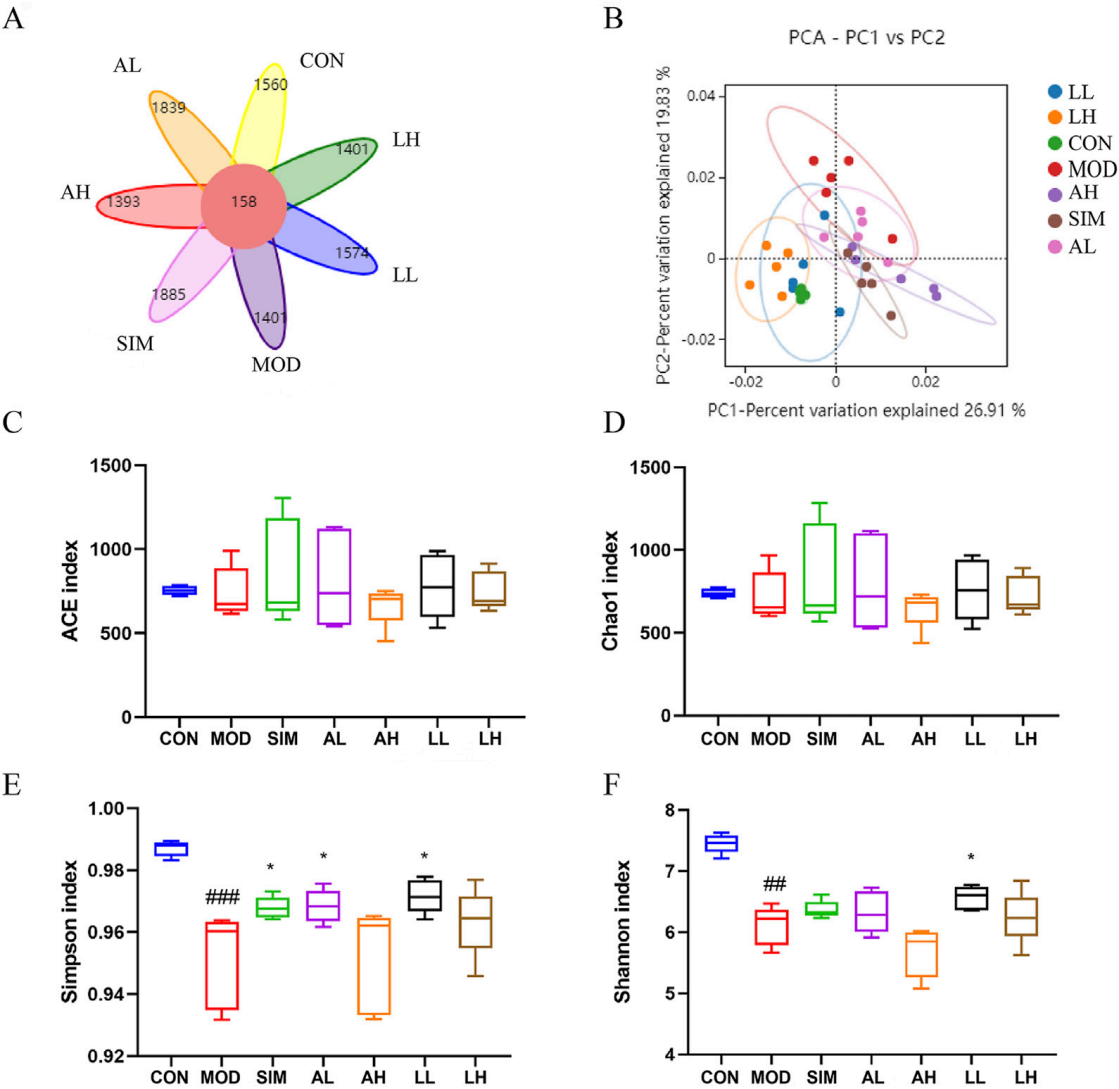
Effects of AR and LC-AR on the mice’s gut microbiota makeup. **(A)** Wayne diagram, **(B)** the PCA plot of the OUT level, **(C)** ACE index, **(D)** Chao 1 index, **(E)** Shannon index, and the **(F)** Simpson index, the data were represented as the mean ± SD, n = 5. Compared with the CON group, ^#^ significant at *p* < 0.05; ^##^ significant at *p* < 0.01; and ^###^ significant at *p* < 0.001; compared with MOD group, ^*^ significant at *p* < 0.05; ^**^ significant at *p* < 0.01; and ^***^ significant at *p* < 0.001.

Gut microbiota represents a vast bacterial community present in the gastrointestinal tract. Jia et al. (2021) argued that a normal gut microbiota is crucial for maintaining lipid metabolism homeostasis. To investigate the impact of a high-fat diet on gut microbiota and whether AR and LC-AR can improve gut microbiota imbalance caused by hyperlipidemia, the bacterial community composition in the gut was analyzed using 16S rRNA sequencing. Based on operational taxonomic units (OTUs), the Venn diagram (Figure 5A) shows an intersection of 158 OTUs among all groups, with the model group having a lower OTU count (1401) compared to the control group (1560). Meanwhile, we applied multivariate statistical techniques to analyze the differences in gut microbiota composition between the groups from a macroscopic perspective. The PCA in Figure 5B demonstrates a clear separation between the control and model groups, with each treatment group tending toward the control group.

Alpha diversity analysis was conducted using ACE, Chao1, Simpson, and Shannon indices as the evaluation criteria (Figures 5C–F). ACE and Chao1 indices represent the abundance of the microbiota, while Simpson and Shannon indices represent the diversity of the microbiota. The ACE and Chao1 indices showed no significant differences among the groups (*p* > 0.05), indicating that the abundance of gut microbiota was affected by neither the high-fat diet nor the treatment with AR and LC-AR. Furthermore, compared to the control group, the Simpson and Shannon indices of the model group significantly decreased (*p* < 0.01). Meanwhile, the Simpson index in the SIM, BL, and FL groups, as well as the Shannon index in the FL group, all showed significant increases compared to the model group (*p* < 0.05). The above results indicate that AR and LC-AR can improve the diversity of gut microbiota to some extent.

### 3.11 Analysis of gut microbiota in mice with hyperlipidemia by species composition and abundance

**FIGURE 6:**
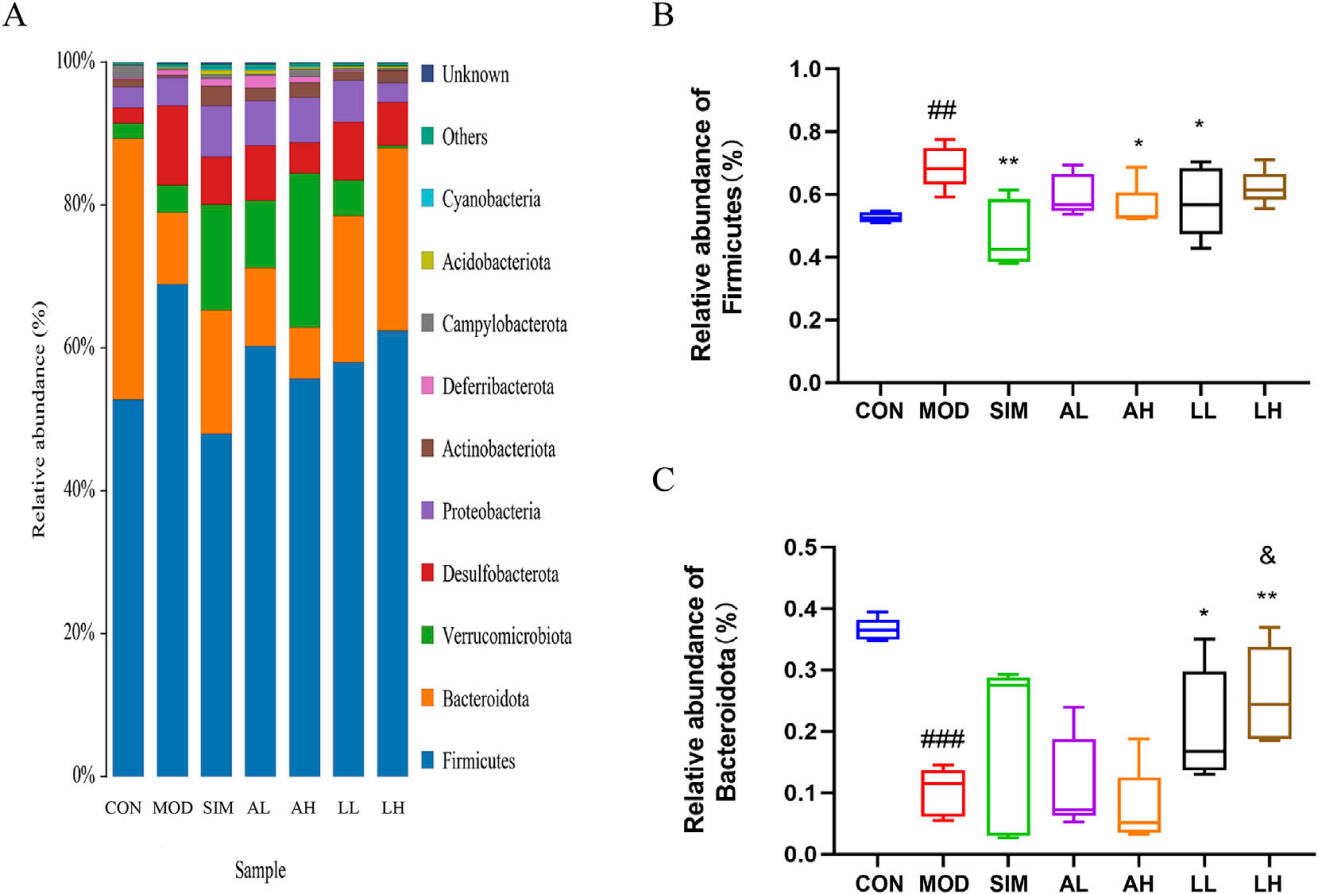
Effects of AR and LC-AR on the relative abundance of gut microbiota at the phylum level. **(A)** Gut microbiota relative abundance at the phylum level, **(B)** Firmicutes relative abundance, and **(C)** Bacteroidota relative abundance. The data were represented as the mean ± SD, n = 5. In comparison to the CON group, the significance levels were as follows: ^#^ significant at *p* < 0.05; ^##^ significant at *p* < 0.01; and ^###^ significant at *p* < 0.001; compared with MOD group, ^*^ significant at *p* < 0.05; ^**^ significant at *p* < 0.01; and ^***^ significant at *p* < 0.001. Compared to the AL group, ^&^ significant at *p* < 0.05.

In microbiology, microbial species are classified into eight hierarchical levels: domain, kingdom, phylum, class, order, family, genus, and species. Based on the OTU analysis, a species analysis was performed at the phylum level to assess the microbial composition within different groups (Figure 6A). In general, bacterial phylum with a relative abundance greater than 0.5% is defined as major phylum, also referred to as the dominant phylum. Firmicutes was evidently the predominant bacterial phylum in all groups. The relative abundance of Firmicutes in the model group significantly increased compared to the control group (*p* < 0.05), while that in the SIM, AH, and LL groups significantly decreased (*p* < 0.05) (Figure 6B).

In addition, Bacteroidota had the second-highest relative abundance in all groups. According to Figure 6C, the relative abundance of Bacteroidota in the model group significantly decreased compared to the control group (*p* < 0.05), while that in the LL and LH groups significantly increased (*p* < 0.05).

AR and LC-AR can modulate gut microbiota homeostasis by downregulating the relative abundance of Firmicutes phylum and upregulating that of Bacteroidota phylum to potentially ameliorate hyperlipidemia induced by a high-fat diet.

**FIGURE 7:**
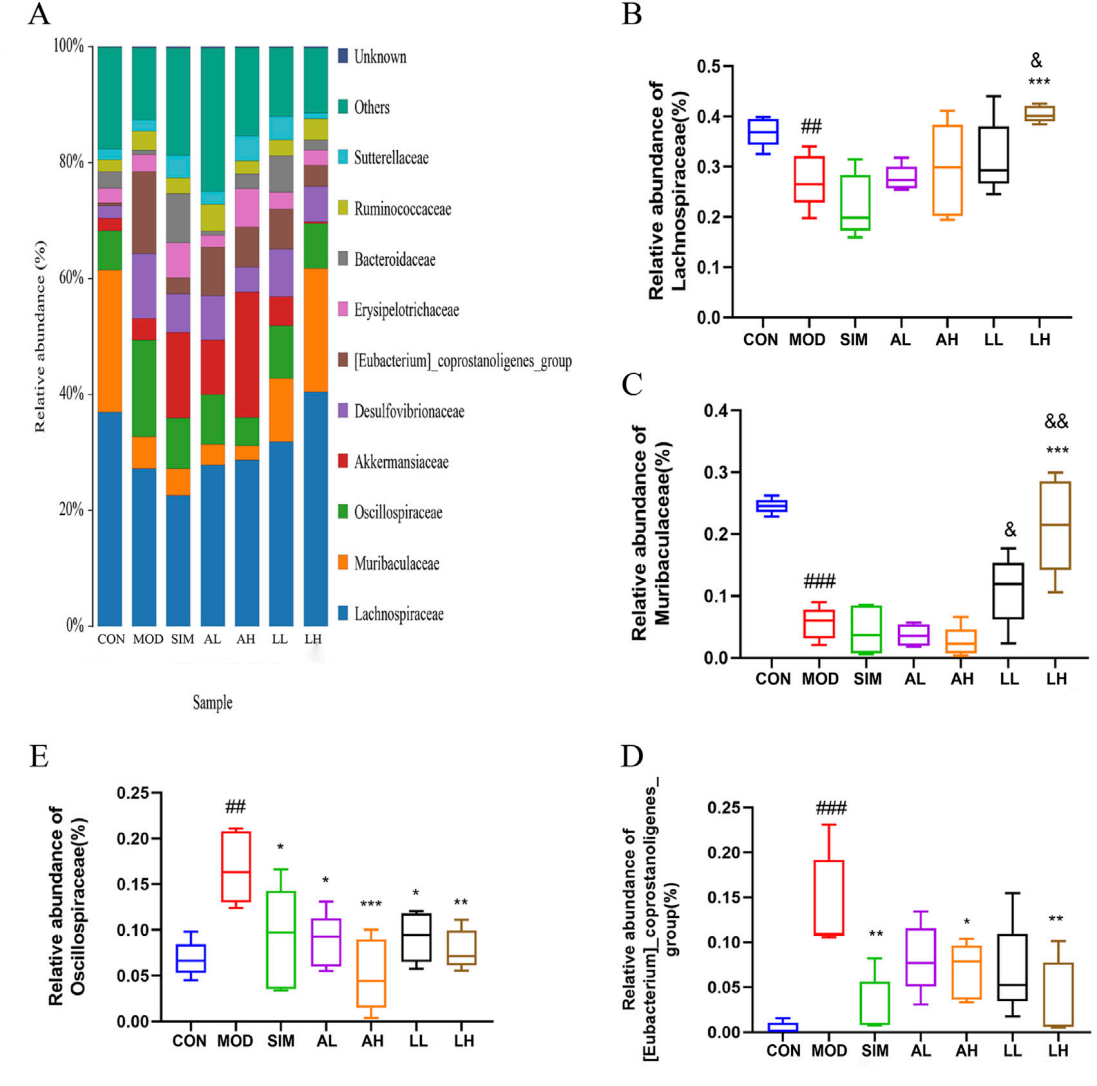
Effects of AR and LC-AR on the relative abundance of gut microbiota at the family level. **(A)**The gut microbiota’s abundance at the family level, **(B)** Lachnospiraceae relative abundance, **(C)** Muribaculaceae relative abundance, **(D)** [Eubacterium]_coprostanoligenes_group relative abundance, and **(E)** Oscillospiraceae relative abundance were expressed as mean ± SD, n = 5. In comparison to the CON group, ^#^ significant at *p* < 0.05; ^##^ significant at *p* < 0.01; ^###^ significant at *p* < 0.001. When compared to the MOD group, ^*^ significant at *p* < 0.05; ^**^ significant at *p* < 0.01; ^***^ significant at *p* < 0.001. Compared to the AL group, ^&^ significant at *p* < 0.05, ^&&^ significant at *p* < 0.01.

We conducted further analysis of the composition of gut microbiota at the family level (Figure 7). In general, a bacterial family with a relative abundance greater than 0.5% is defined as a major family, also referred to as a dominant family. Lachnospiraceae, Muribaculaceae, Oscillospiraceae, and [Eubacterium]_coprostanoligenes_group are dominant bacterial families in the microbial composition of each group (Figure 7A). According to Figures 7B, C, the relative abundances of Lachnospiraceae and Muribaculaceae in the model group significantly decreased compared to the control group (*p* < 0.05), while those in the LL and LH groups exhibited varying degrees of increase. Additionally, the relative abundances of Oscillospiraceae and [Eubacterium]_coprostanoligenes_group in the model group significantly increased compared to the control group (*p* < 0.05), whereas those in the SIM, AL, AH, LL, and LH groups exhibited varying degrees of reduction (Figures 7D, E).

AR and LC-AR can modulate gut microbiota homeostasis by downregulating the relative abundance of Oscillospiraceae and [Eubacterium]_coprostanoligenes_group and upregulating that of Lachnospiraceae and Muribaculaceae at the family level.

### 3.12 Analysis of gut microbiota in mice with hyperlipidemia by significant difference and correlation

**FIGURE 8:**
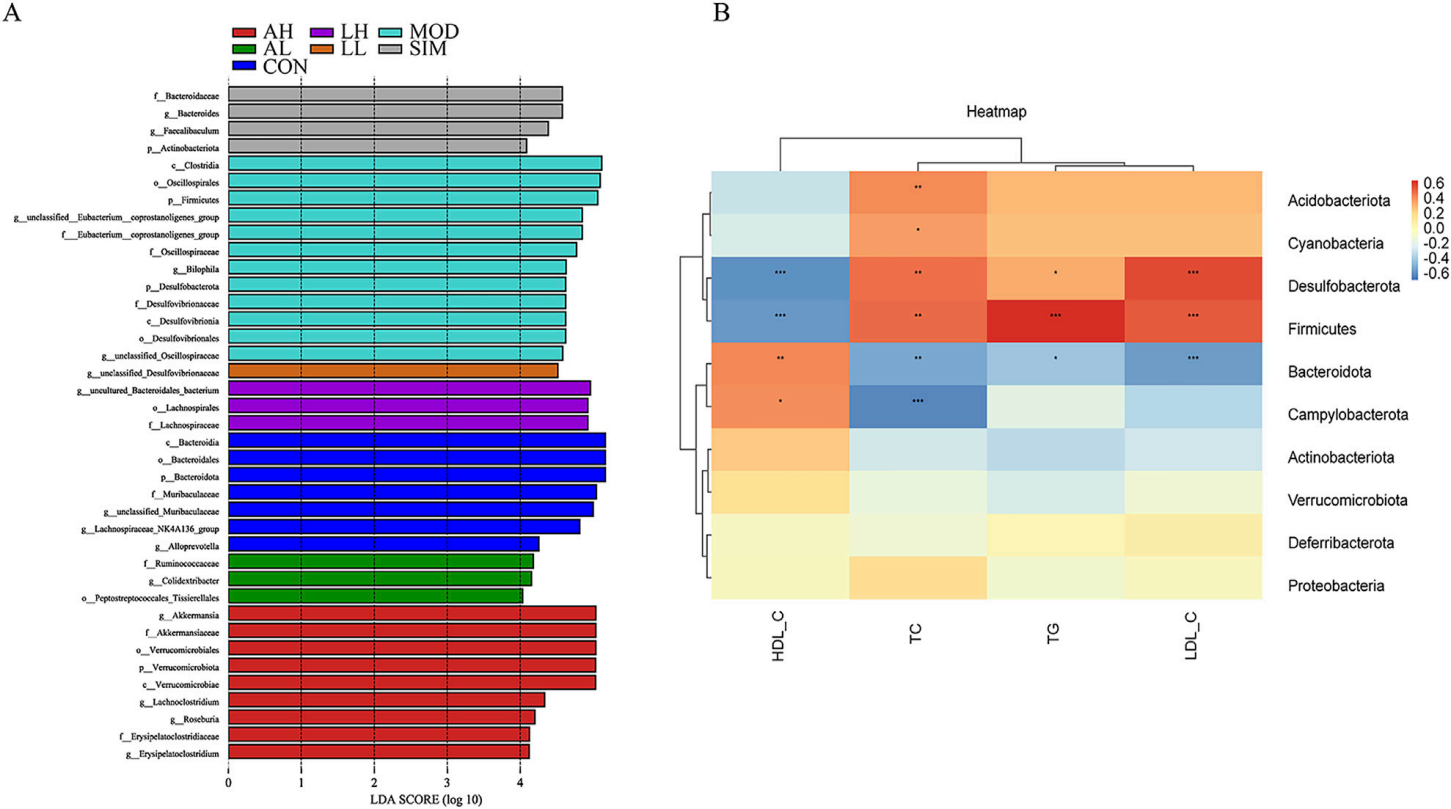
Effects of AR and LC-AR on significant differences in gut microbiota and correlation analysis between lipid levels and gut microbiota. **(A)** Significant difference analysis based on LEfSe (Log LDA > 4.0); **(B)** Correlation Analysis Heatmap Based on Spearman’s Correlation. The intensity of colors represents the degree of association between the gut microbiota and lipid levels, with blue indicating a negative connection and red indicating a positive correlation. ^*^ significant at *p* < 0.05, ^**^ significant at *p* < 0.01, and ^***^ significant at *p* < 0.001.

The linear discriminant analysis algorithm-based LDA effect size technique was used to analyze microbiota species exhibiting significant differences among different groups (Figure 8A). Bacteroidota, Bacteroidia, Bacteroidales, and Muribaculaceae were the major significantly different microbial species distinguishing the control group from the other groups. In contrast, f_Eubacterium_coprostanoligenes_group, Oscillospiraceae, Clostridia, and Firmicutes were the significantly different microbial species in the model group, which indicates the pathogenic mechanisms of hyperlipidemia. Additionally, Lachnospiraceae and g_unclassified_desulfovibrionaceae were identified as significantly different microbial species in the LH and LL groups, respectively. These results indicate significant species variations among the groups.

To explore in depth the potential link between hyperlipidemia and gut microbiota, we conducted a correlation analysis of selected species at the phylum level of the gut microbiome and lipid levels (Figure 8B). Based on the color variations in the correlation heatmap, Acidobacteriota and Cyanobacteria were significantly positively correlated with TC (*p* < 0.05, *p* < 0.01). Both Desulfobacterota and Firmicutes exhibited a significant negative correlation with HDL-C and a significant positive correlation with TC, TG, and LDL-C (*p* < 0.05, *p* < 0.01). Campylobacterota and Bacteroidota were both significantly positively correlated with HDL-C, and Bacteroidota was significantly negatively correlated with TC, TG, and LDL-C. However, Campylobacterota was only significantly negatively correlated with TC (*p* < 0.05, *p* < 0.01). Overall, Acidobacteriota, Cyanobacteria, Desulfobacterota, Firmicutes, Campylobacterota, and Bacteroidota are pivotal microbial species through which AR and LC-AR improve the lipid levels by modulating gut microbiota.

## 4 Discussion

Hyperlipidemia is generally linked to metabolic disturbances, and consuming a diet rich in fats and cholesterol may contribute to the condition (Zhang Q. et al., 2020). Imbalances in gut microbiota and disruptions in lipid levels are common complications of hyperlipidemia (Tong et al., 2018). Modulating the gut microbiota equilibrium and improving the lipid levels can be the primary strategies for alleviating hyperlipidemia.

Inhibiting the activity of PL and cholesterol esterase CEase can delay the digestion and absorption of a high-fat diet (Chatatikun and Kwanhian, 2020). Toma et al. (2014) found that the potential of Moringa stenopetala leaves to lower lipid levels is attributable to their significant inhibition of PL and CEase activities. Therefore, PL and CEase are frequently used to assess the *in vitro* lipid-lowering effects of drugs. To identify strains that may be more suitable for improving hyperlipidemia, we assessed the *in vitro* hypolipidemic activity of various *Lactobacillus* fermenting AR and found that the AR fermentation by LC exhibited superior inhibitory effects on PL and CEase compared to other strains. Kapila and Sinha, 2006 found that LC, exhibiting relatively strong *in vitro* antioxidant activity, also demonstrated effective cholesterol-lowering effects in mice. Qian et al. (2019) indicated that LC can improve the lipid metabolism levels in mice with hyperlipidemia. There was a significant increase in the total phenolic content and *in vitro* antioxidant activity of LC-AR, accompanied with a notable correlation. This result was foreseeable, as the cellulase produced by LC can hydrolyze the cellulose of AR, releasing more phenolic compounds, which exhibit significant *in vitro* antioxidant activity (El-Saadony et al., 2021). Additionally, LC itself possesses strong antioxidant capabilities (Kapila and Sinha, 2006). Natural antioxidants seem to have a certain potential for improving liver damage caused by oxidative stress (Li W. et al., 2018), further validating the effectiveness of selecting LC as the fermentation strain. In fact, flavonoids and polysaccharides also exhibit potent *in vitro* antioxidant activity (Li Z. et al., 2018; Gandhi et al., 2020), providing new insights for the related research.

The metabolites of fermentation are the final products of the fermentation process, and the composition of metabolite species determines their potential to play a specific crucial role within the organism. In this study, untargeted metabolomics was employed to conduct a comprehensive analysis of the changes in the species and levels of metabolites prior to and following fermentation. Among the 198 significantly different metabolites, there was a significant upregulation in the levels of 8 phenolic compounds (such as epinephrine, genistein, apigenin, gallic acid, and eriodictyol), 20 amino acids and their derivatives (such as L-Glutamic acid, L-Serine, and L-Isoleucine), and 9 fatty acids [such as 3-(methylthio)propionic acid, and palmitoleic acid]. Figure 1D shows that the metabolite with the highest relative abundance in both the AR and LC-AR groups is epinephrine. Hormone-sensitive triglyceride lipase is a neutral lipase and a rate-limiting enzyme in fat breakdown, which is capable of hydrolyzing TGs and other lipid and water-soluble substrates. There was a report that exogenous epinephrine can increase the activity of neutral lipase in rat soleus muscle *in vitro* (Langfort et al., 1999). Similarly, Prats et al. (2006) demonstrated that exogenous epinephrine can reduce intramuscular TGs and intramuscular lipid droplets in rat soleus muscle. Additionally, multiple studies have demonstrated that dietary phenolic compounds, such as genistein (Tang et al., 2015), apigenin (Xu et al., 2021), gallic acid (Huang et al., 2018), and eriodictyol (Kwon and Choi, 2019), can improve symptoms associated with hyperlipidemia in rats or mice, including lipid level abnormalities, liver lipid accumulation, and liver damage. In particular, eriodictyol exhibits a significant protective effect against liver injury (Zheng et al., 2023).

Among the 20 amino acids and their derivatives, one of the precursors of glutathione, the L-isomer of glutamic acid (L-Glutamic acid, as shown in Figure 1D), shows a significant increase in content after fermentation. Salyha and Salyha (2018) pointed out that L-Glutamic acid can alleviate or inhibit chlorpyrifos-induced oxidative stress in rats. The abundance of L-Glutamic acid in LC-AR seems to support its superior performance over AR in vitro antioxidant experiments. Interestingly, the experiments conducted by Sim et al. (2015) demonstrated that supplementing L-Serine can reduce neutral lipid accumulation and TG concentration in the livers of alcohol-induced fatty liver mice and rats. Additionally, Niu et al. (2023) found that empagliflozin can accelerate the clearance of toxic lipids in non-alcoholic fatty liver mice by upregulating L-Isoleucine levels, thereby alleviating liver damage caused by lipotoxicity. Among the 9 fatty acids, 3-(methylthio) propanoic acid is an intermediate in the metabolism of methionine and a type of fatty acid. Sugiyama et al. (1986) found that supplementing 3-(methylthio) propanoic acid can improve liver lipid levels and plasma cholesterol levels in rats fed a high-cholesterol diet. Palmitoleic acid is a monounsaturated fatty acid. The experiments conducted by Yang Z. H. et al. (2019) demonstrated that the dietary supplementation of palmitoleic acid can improve the plasma levels of LDL-C receptor-deficient mice (TC, TGs, free cholesterol, and phospholipids) and the lipid/lipoprotein profile in the liver. Similarly, the expression levels of mRNA associated with fatty acid synthesis, such as SREBP1c, FASN, and SCD1 mRNA, showed a significant downregulation after being supplemented with palmitoleic acid. Additionally, there was an upregulation in the expression levels of mRNA related to bile acid synthesis and Cyp7a1 mRNA, promoting cholesterol metabolism.

Body weight and liver weight increase are apparent manifestations of hyperlipidemia (Mudgil et al., 2021). Simultaneously, the abnormalities in TC, TG, and LDL-C elevation, along with an abnormal decrease in HDL-C, are common characteristics of hyperlipidemia (Yang et al., 2022). Dyslipidemia can elevate the levels of inflammatory cytokines, leading to the occurrence of inflammatory reactions (Alzahrani et al., 2022). Additionally, proinflammatory cytokines (TNF-α, IL-6) can stimulate TG synthesis and secretion and activate cholesterol synthesis, further affecting the body’s lipid levels (Jung and Choi, 2014). Moreover, an imbalance in the lipid levels will gradually affect the normal functioning of liver cells (Chen et al., 2020). We found that supplementation with LC-AR reduced body weight and liver weight in hyperlipidemic mice. Regarding lipid levels, it decreased the levels of TC, TG, and LDL-C while increasing the level of HDL-C. Meanwhile, the proinflammatory cytokines TNF-α and IL-6 were effectively suppressed. Additionally, LC-AR improved the levels of liver damage markers AST and ALT in the blood, as well as hepatic tissue degeneration at the histological level. Thus, it can be seen that LC-AR performs well in improving the underlying abnormal indicators of hyperlipidemia. Diez-Ozaeta and Astiazaran (2022) emphasized the health benefits of fermented foods and the promising prospects for development in the context of hyperlipidemia. According to Yan et al. (2022), probiotic-fermented rice buckwheat alleviated lipid abnormalities in hyperlipidemic mice.

Cholesterol level is closely related to hyperlipidemia, and the factors influencing cholesterol level include the absorption, synthesis, transport, and excretion of cholesterol. We found that LC-AR can inhibit the expression levels of 3-hydroxy-3-methylglutaryl coenzyme A (HMG-CoA) reductase (HMGCR), sterol-regulatory element-binding proteins (SREBP-2) in the liver, and Niemann-Pick C1-like 1 (NPC1L1) mRNA in the small intestine while promoting the expression of Cholesterol 7-alpha hydroxylase (CYP7A1) mRNA (in the liver). HMGCR is a rate-limiting enzyme in cholesterol biosynthesis, and its activation leads to increased cholesterol synthesis, which may result in hepatic cholesterol accumulation and hypercholesterolemia (Li et al., 2019). CYP7A1 is a rate-limiting enzyme in the bile acid synthesis pathway that promotes the conversion of cholesterol to bile acid and reduces cholesterol accumulation (Li et al., 2021). SREBP-2 primarily regulates the expression of cholesterol synthesis of mRNA (Song et al., 2015). NPC1L1 is a highly expressed transmembrane protein in mammalian small intestine epithelial cells and plays a crucial role in cholesterol absorption (Zhang et al., 2022). NPC1L1 inhibitors have been shown to lower the plasma levels of TGs and cholesterol, thereby regulating lipid metabolism (Tavares et al., 2020). Yang W. S. et al. (2019) found that Chitooligosaccharide tablets can regulate serum cholesterol synthesis levels by upregulating CYP7A1 and downregulating HMGCR and SREBP-2 mRNA expression. Therefore, SREBP-2, HMGCR, CYP7A1, and NPC1L1 mRNA can be effective targets for reducing cholesterol accumulation in the body.

The composition and function of gut microbiota are associated with the host’s energy metabolism and health (Cheng et al., 2022). Most research considers the dysbiosis of gut microbiota to be another important factor leading to hyperlipidemia. Hyperlipidemia typically causes an increase in the ratio of Firmicutes to Bacteroidota (Jiang et al., 2021; De et al., 2011). Similar to our work, there was an increase in the relative abundance of Firmicutes and a decrease in that of Bacteroidota at the phylum level. After supplementation with LC-AR, there was a certain degree of reversal of the trend of an increased Firmicutes/Bacteroidota ratio. Zhang W. et al. (2022) demonstrated that bacterial cellulose reduced the Firmicutes/Bacteroidota relative abundance ratio and improved the activity of gut microbiota in hyperlipidemic mice. Also, a decrease in the ratio of Firmicutes to Bacteroidetes in the gut microbiota is found significantly correlated with various inflammatory markers (Zhang et al., 2023). Therefore, interventions that restrict the elevation of Firmicutes or promote the reduction of Bacteroidota contribute to the treatment of hyperlipidemia.

Additionally, at the family level, supplementation with LC-AR increased the relative abundance of Lachnospiraceae and Muribaculaceae while decreasing the relative abundance of [Eubacterium]_coprostanoligenes_group and Oscillospiraceae. Reportedly, both Lachnospiraceae and Muribaculaceae can produce short-chain fatty acids (SCFAs) (Soares et al., 2021; Ye et al., 2021). Previous research has indicated that butyrate salts, a type of SCFAs produced by Lachnospiraceae, can maintain the integrity of the intestinal barrier and suppress inflammation in mice (Ma et al., 2020). Niu et al. (2022) suggested that Lachnospiraceae can regulate the absorption of nutrients in the gut and inhibit the growth and proliferation of pathogenic microorganisms. However, Oscillospiraceae is significantly associated with lipid abnormalities and systemic inflammatory responses (Farzam, 2022). Related studies have suggested that the Eubacterium_coprostanoligenes_group is positively correlated with lipid levels, such as TC, TG, and LDL-C (Lei et al., 2023). Overall, at the phylum and family taxonomic levels, LC-AR demonstrated the potential to ameliorate hyperlipidemia resulting from the dysbiosis of gut microbiota.

According to our research findings, the fermentation of LC enhances the effectiveness of AR in improving hyperlipidemia. The main mechanism through which LC-AR exerts its anti-hyperlipidemic effects is the regulation of lipid levels and cholesterol-related mRNA, as well as the gut microbiota. To the best of our knowledge, this is the first report related to the LC fermented AR. However, this research is still in the exploratory phase, with the experimental model limited to animal experiments, and it lacks comparisons with existing drugs such as statins and fibrates, or with the efficacy of traditional fermented Chinese medicines. Further clinical trials are needed to evaluate its potential as an alternative to current treatment options. Additionally, the specific active components in LC-AR and the exact mechanisms underlying its antihyperlipidemic effects require further investigation.

## 5 Conclusion

This study highlights the potential of LC-AR as a promising supplement for improving hyperlipidemia. The selection of LC was based on *in vitro* lipid-lowering experiments, and the combination with AR demonstrated higher polyphenol content and superior antioxidant activity compared to AR alone. Simultaneously, the variations in metabolite levels revealed by untargeted metabolomics provided support for the feasibility of the experiment. LC-AR showed favorable effects in regulating the lipid levels (TC, TG, LDL-C, and HDL-C) and liver injury markers (AST and ALT), proinflammatory cytokine levels (TNF-α and IL-6) and regulating the expression of cholesterol-related mRNA (HMGCR, SREBP-2, CYP7A1, and NPC1L1 mRNA). Additionally, LC-AR exhibited positive changes in liver tissue pathology and alterations in gut microbiota species. These findings are significant as they offer a new perspective on the study of traditional Chinese medicine’s effects on hyperlipidemia. They contribute to the theoretical understanding of the transformation and utilization of LC and AR, and provide a scientific basis for considering LC-AR as a potential therapeutic supplement and drug raw materials for hyperlipidemia.

## Data Availability

The 16s rRNA data presented in the study has been deposited in the NCBI (SRA) repository, accession number PRJNA1174375. Further inquiries can be directed to the corresponding authors.
