# Supplementary material for: Effects of fermented Arctium lappa L. root by Lactobacillus casei on hyperlipidemic mice
Source: Front Pharmacol. 2024 Oct 28;15:1447077. doi: 10.3389/fphar.2024.1447077 (PMC11551023; doi:10.3389/fphar.2024.1447077)
Supplement: Supplementary file 1 [file DataSheet1.ZIP › Table S1.docx]

**Supplementary Table S1.** Real-time RT-PCR primers utilized.

| **Primer name** | **Forward (5 ' -3 ')** | **Reverse (5 ' -3 ')** |
| --- | --- | --- |
| Hmgcr | TGCCTGGATGGGAAGGAGTA | AAATCTGCTGGTGCTATCAAAGG |
| SREBP-2 | TCTGAGGAAGGCCATTGATTACA | CAAGTCCACATCACTGTCCACCA ^1^ |
| Cyp7a1 | AGGTCTCTGAACTGATCCGTCTACG | GCGTCTTAGCCTTCTCCATGTCATC |
| NPC1L1 | CCTTTGTGGTCTGCTACCTCCTA | CTGCCGTGACAAGGTTGATGAG |
| GAPDH | CCTCGTCCCGTAGACAAAATG | TGAGGTCAATGAAGGGGTCGT |
